# Supplementary material for: Lifestyle Related Cancer Risk and Protective Behaviors Vary among a Convenient Sample of Physically Active, Young-to-Middle-Aged Adults 18–49
Source: Int J Environ Res Public Health. 2023 Jul 6;20(13):6305. doi: 10.3390/ijerph20136305 (PMC10341878; doi:10.3390/ijerph20136305)
Supplement: Supplementary file 1 [file ijerph-20-06305-s001.zip › ijerph-2410304-supplementary.pdf]

Supplementary Tables

Table S1

Cancer-Related Risk and Protective Behaviors among Physically Active, Young-to-Middle Aged Adults 18-49. Variables were to create dichotomous healthy or unhealthy behaviors in the study.

Lifestyle Behavior		Study Sample n (%)
Physical Activity		
	0 days per week	27 (3.1%)
	1 day per week	55 (6.3%)
	2 days per week	104 (11.9%)
	3 days per week	207 (23.8%)
	4 days per week	152 (17.5%)
	5 days per week	160 (18.4%)
	6 days per week	61 (7.0%)
	7 days per week	102 (11.7%)
	Don’t Know	3 (0.3%)
Fruit Consumption		
	None	63 (7.2%)
	1/2 cup or less	169 (19.4%)
	1/2 to 1 cup	195 (22.4%)
	1 to 2 cups	247 (28.4%)

	2 to 3 cups	119 (13.7%)
	3 to 4 cups	41 (4.7%)
	4 or more cups	36 (4.1%)
Vegetable Consumption		
	None	71 (8.4%)
	1/2 cup	150 (17.2%)
	1/2 to 1 cup	183 (21.0%)
	1 to 2 cups	232 (26.6%)
	2 to 3 cups	122 (14.0%)
	3 to 4 cups	75 (8.6%)
	4 or more cups	39 (4.5%)
Current Cigarette Smoking Status		
	Every day	118 (13.6%)
	Some days	84 (9.7%)
	Not at all	665 (76.7%)
Current E-cigarette and "Vape" Use Status		
	Every day	49 (5.7%)
	Some days	100 (11.6%)

	Not at all	715 (82.8%)
Chewing Tobacco, Snuff or Snus		
	Every day	26 (3.0%)
	Some days	46 (5.3%)
	Not at all	795 (91.7%)
Any Past 30-Day Alcohol Use		
	Yes	676 (77.4%)
	No	197 (22.6%)
Binge Drinking		
Yes		
	Men	273 (61.9%)
	Women	248 (62.3%)
No		318 (37.9%)
	Men	168 (38.1%)
	Women	150 (37.7%)
Use of Sun Protection		
	Always	90 (10.7%)
	Most of the time	189 (23.4%)
	Sometimes	249 (29.5%)
	Rarely	197 (23.3%)
	Never	101 (12.0%)

Don’t stay outside for more than one hour on warm sunny days	16 (1.9%)
Don’t go outside at all on warm sunny days	3 (.4%)
Routine Doctor Visits	
Yes	672 (79.5%)
No	173 (20.5%)

Table S2

Cumulative healthy behaviors present and all combinations with ≥3 Days of physical activity stratified by sex				
		Overall n (%)	Men (n)%	Women (n)%
0	No healthy behaviors reported	32 (4.4%)	14 (3.6%)	18 (5.4%)
1	Physical activity only (P)	61 (8.4%)	41 (10.4%)	20 (6.0%)
	Fruit and Vegetable Consumption only (D)	4 (0.5%)	2 (0.5%)	2 (0.6%)
	No current tobacco use only (S)	38 (5.2%)	19 (4.8%)	19 (5.7%)
	No binge drinking only (A)	8 (1.1%)	5 (1.3%)	3 (0.9%)
	Safe/Optimal sun protection use only (SP)	12 (1.6%)	8 (2.0%)	4 (1.2%)
	Total	123 (16.8%)	75 (19.0%)	48 (14.3%)
2				
	P + D	33 (4.5%)	23 (5.8%)	10 (3.0%)
	P + S	122 (16.7%)	56 (14.2%)	66 (19.6%)
	P +A	29 (4.0%)	20 (5.1%)	9 (2.7%)
	P + SP	44 (6.0%)	26 (6.6%)	18 (5.4%)
	Total	228 (31.2%)	125 (31.7%)	103 (30.7%)
3				
	P + D + S	38 (5.2%)	18 (4.6%)	20 (6.0%)
	P + D + A	3 (0.4%)	3 (0.8%)	0 (0%)
	P + D + SP	18 (2.5%)	10 (2.5%)	8 (2.4%)
	P + S + A	103 (14.1%)	60 (15.2%)	43 (12.8%)
	P + S + SP	53 (7.3%)	28 (7.1%)	25 (7.4%)
	P + A + SP	13 (1.8%)	8 (2.0%)	5 (1.5%)
	Total	228 (31.2%)	127 (32.2%)	101 (30.1%)

4				
	P + D + S + A	19 (2.6%)	9 (2.3%)	10 (3.0%)
	P + D + S + SP	22 (3.0%)	12 (3.0%)	10 (3.0%)
	P + S + A + SP	45 (6.2%)	19 (4.8%)	26 (7.7%)
	P + A + SP + D	4 (0.5%)	3 (0.8%)	1 (0.3%)
	Total	90 (12.3%)	43 (10.9%)	47 (14.0%)
5				
	P + D + S + A + SP	29 (4.0%)	10 (2.5%)	19 (5.7%)
Overall		730	394	336

Number of healthy lifestyle behavior within mutually exclusive combinations of physical activity.

Proportions are column percentages.

Observations with any missing health behaviors were excluded from the analysis.

Table S3

Cumulative unhealthy behaviors present and all combinations with $\leq$2 physical inactivity stratified by sex				
		Overall (n)%	Men (n)%	Women (n)%
0	No unhealthy behaviors reported	29 (9.9%)	10 (7.5%)	19 (11.9%)
1	Physical inactivity only (P)	1 (0.3%)	1 (0.8%)	0 (0%)
	No Fruit and Vegetable Consumption only (D)	45 (15.4%)	19 (14.3%)	26 (16.4%)
	Current tobacco use only (S)	4 (1.4%)	3 (2.3%)	1 (0.6%)
	Binge drinking only (A)	22 (7.5%)	12 (9.0%)	10 (6.3%)
	Unsafe/Suboptimal sun protection use only (SP)	19 (6.5%)	9 (6.8%)	10 (6.3%)
	Total	91 (31.2%)	44 (33.1%)	47 (29.6%)
2				
	P + D	14 (4.8%)	7 (5.3%)	7 (4.4%)
	P + S	0 (0%)	0 (0%)	0 (0%)
	P + A	2 (0.7%)	0 (0%)	2 (1.3%)
	P + SP	3 (1.0%)	0 (0%)	3 (1.9%)
	Total	19 (6.5%)	7 (5.3%)	12 (7.5%)
3				
	P + D + S	1 (0.3%)	1 (0.8%)	0 (0%)

	P + D + A	15 (5.1%)	4 (3.0%)	11 (6.9%)
	P + D + SP	34 (11.6%)	15 (11.3%)	19 (11.9%)
	P + S + A	1 (0.3%)	1 (0.8%)	0 (0%)
	P + S + SP	2 (0.7%)	2 (1.5%)	0 (0%)
	P + A + SP	6 (2.1%)	1 (0.8%)	5 (3.1%)
	Total	59 (20.2%)	24 (18.0%)	35 (22.0%)
4				
	P + D + S + A	12 (4.1%)	8 (6.0%)	4 (2.5%)
	P + D + S + SP	8 (2.7%)	5 (3.8%)	3 (1.9%)
	P + S + A + SP	4 (1.4%)	2 (1.5%)	2 (1.3%)
	P + A + SP + D	38 (13.0%)	19 (14.3%)	19 (11.9%)
	Total	62 (21.2%)	34 (25.6%)	28 (17.6%)
5				
	P + D + S + A + SP	32 (11.0%)	14 (10.5%)	18 (11.3%)
Overall		292	133	159

Number of unhealthy lifestyle behavior within mutually exclusive combinations of physical activity.

Proportions are column percentages.

Observations with any missing health behaviors were excluded from the analysis.
